# Clinicians’ practices and attitudes toward depression assessment: a cross-sectional survey study

**DOI:** 10.1186/s40359-026-05036-w

**Published:** 2026-08-19

**Authors:** Damon Navandi, Veerle C. Eijsbroek, Clara Wiebel, Aaron Marker, Katarina Kjell, Marc L. Molendijk, Elizabeth C. Stade, Oscar N. E. Kjell

**Affiliations:** 1 Lund University, Lund, Sweden; 2 Vanderbilt University, Nashville, TN USA; 3 Leiden University, Leiden, Netherlands; 4 Stanford University, Stanford, CA USA

**Keywords:** Clinical assessment, Depression, Structured methods, Statistical models, Artificial intelligence

## Abstract

Accurate psychological evaluation is crucial for early detection, intervention and evaluation of mental health disorders. The validity and reliability of psychological evaluation methods have been systematically researched for many decades. However, assessment methods used by mental health professionals in clinical settings have not consistently been empirically documented. This study examines the extent to which standardized assessment methods are used in clinical settings, and clinicians’ attitudes toward these practices. Clinicians (*N* = 585) from the U.S. (25%), the U.K. (28%), the Netherlands (18%), Sweden (22%), and other countries (7%) completed an online survey reporting their depression assessment methods. Clinicians reported using many different *data collection methods* (e.g., unstructured clinical interviews [83%], rating scales [83%]) in various combinations. A majority (80%) primarily employed clinical data combination methods, as opposed to statistical *data combination methods* (11%). Although they were confident in their assessments, the time to carry out assessments varied widely (*M* = 87 [*SD =* 108] minutes). 77% agreed on the importance of standardized, valid, and reliable assessments, and 43% reported interest in receiving AI decision-support. We did not find many significant cross-cultural differences. A majority of clinicians have not fully adopted standardized assessment methods. Predominant reliance on clinical judgment suggests a disconnect between best practices supported by research and everyday clinical practice.

**Supplementary Information:**

The online version contains supplementary material available at 10.1186/s40359-026-05036-w.

## Introduction

**Fig. 1:**
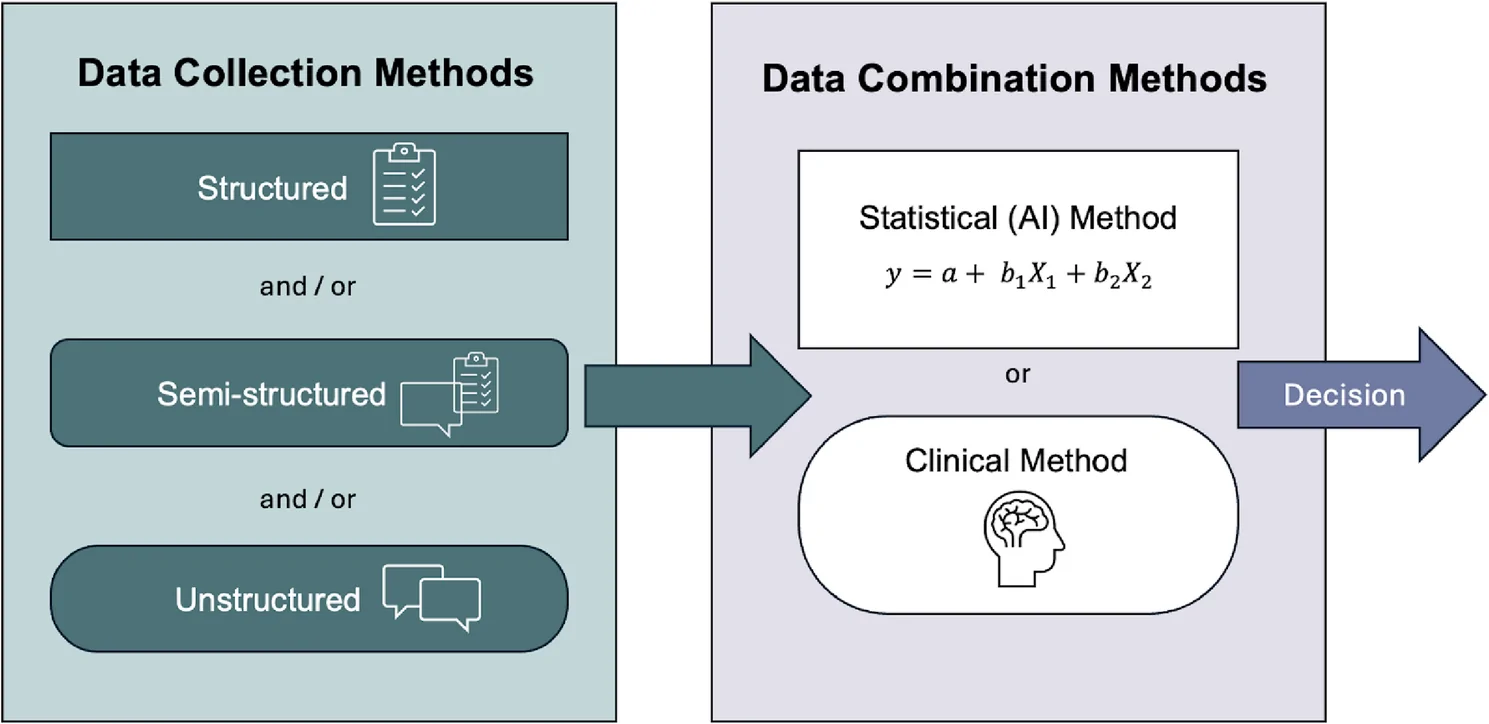
Overview of depression assessment methods, including data collection and data combination methods. *Note*. A data combination method does not necessarily require information from multiple data collection methods. For example, responses to a single rating scale can be combined and interpreted using either a statistical model (e.g., an aggregation algorithm or established cut-off score) or a clinical method, in which individual items are interpreted using clinical judgment and experience. Likewise, data obtained from a clinical (unstructured) interview can be incorporated into a statistical model (e.g., response content, response times, or observed behaviors)

Accurate assessment of mental health disorders is crucial for early detection and effective treatment planning. Inaccurate assessment of the type and severity of mental disorders can have significant negative impacts on people’s lives, highlighting the importance of precise assessment methods for effective prevention, early detection, and appropriate treatment [1–3]. There are many different methods used to assess mental health problems, including various *data collection methods* such as interviews and rating scales, as well as various *data combination methods*, i.e., methods for evaluating, integrating, and interpreting different sources of information (Fig. 1) [4, 5]. We distinguish between *data collection methods* (i.e., how information is gathered, such as interviews or rating scales) and *data combination methods* (i.e., how information is integrated and interpreted, such as clinical judgment or statistical models). These represent conceptually distinct stages in the assessment process.

The validity and reliability of depression assessment methods have been extensively studied [6, 7], with some methods being more reliable and valid than others are. However, less is known about the practices of mental health professionals, and the available evidence suggests a discrepancy between guidelines on standards of practice and those on clinical practice [8]. This study aims to gain insights into the extent to which standardized assessment methods are used by mental health professionals, as well as their attitudes toward these methods, which is critical for identifying risks and challenges in current assessment practices and any implementation of new assessment methods. Our study may also add to the discourse on integrating artificial intelligence-based (AI) decision-support systems into assessment processes [9–11]. A growing body of research supports the use of AI in mental health care, demonstrating its ability to improve the accuracy and efficiency of diagnostic assessments [10, 12, 13]. Given its promising applications and increasing capabilities, we focus on AI as a key component of the statistical methods approach in the following sections.

### Data collection methods

Data collection methods range from *unstructured* to *structured* [14]. *Unstructured data collection methods* include *unstructured clinical interviews*, which allow clinicians to explore patients’ issues freely and adjust the direction of the assessment on the basis of patient’s responses and behaviors without predetermined questions. *Structured data collection methods* are highly organized and follow a predetermined format, ensuring that the same questions are asked of each person. Structured methods aim to yield more reliable and comparable results and are especially well suited for assessing a set of validated criteria for a disorder or clinical phenomenon (e.g., evaluating whether a patient meets a diagnostic manual’s criteria for a major depressive episode). Examples include rating scales and structured clinical interviews, as well as semi-structured clinical interviews where questions are predetermined, but the interviewer is allowed to ask additional questions or exclude ones to adjust the sourcing of information on a case-to-case basis. The interviews were conducted by clinicians, while for the rating scales, some were clinician-rated, and others were self-reported.

### Data combination methods

Two primary methods for evaluating, integrating, and interpreting different sources of information have been identified for depression assessment: the *clinical* method and the *statistical* method [6, 15, 16]. *The clinical data combination method* is based on human judgment. It is an integrative judgment process in which clinicians synthesize multiple sources of information using implicit, experience-based reasoning rather than formalized rules or algorithms. In this method, information (e.g., symptoms, patient history, behavioral observations) is selectively attended to, weighted, and combined based on clinical expertise, heuristics, and contextual interpretation. The weighting and integration process is typically not explicitly specified, may vary from time to time for the same clinician or across clinicians and is not constrained by a predefined model. This process may involve both intuitive (automatic) and analytical (deliberate) components of decision-making. In contrast, *the statistical data combination method* makes use of a statistical model that is based on established relationships between the different sources of information and the outcome of interest. This can, for example, involve the clinician entering client data into formulas, actuarial tables, or charts [15, 16]. To establish relationships among variables, statistical models may be constructed via relatively simple statistical methods such as linear or logistic regression. It can also involve more complex statistical techniques, such as AI, which includes machine learning and natural language processing. Statistical data combination methods can, for example, be used for screening, comprehensive diagnostic assessment support, treatment personalization, or symptom monitoring [12, 17].

### Final assessment and decision-making

In the data combination phase, both the clinical and the statistical data combination methods can be used at different stages as described in the Brunswick Lens Model [18–20]. The Brunswik Lens Model provides a framework for understanding how individuals make judgments under uncertainty by integrating multiple information cues. In this model, environmental cues (e.g., symptoms, test scores, behavioral observations) are probabilistically related to an outcome of interest (e.g., presence of depression). The clinician perceives and weighs these cues and combines them into a judgment.

The model distinguishes between (a) the *ecological validity* of cues (i.e., how strongly cues are actually related to the outcome) and (b) the *cognitive strategy* of the decision-maker (i.e., how cues are subjectively weighted and combined). The correspondence between judgments and actual outcomes reflects decision accuracy.

In the context of the present study, data collection methods correspond to the acquisition of cues (e.g., interviews, rating scales), whereas data combination methods reflect how these cues are integrated. Clinical data combination methods rely on implicit, experience-based weighting of cues, whereas statistical methods formalize this process using explicit models. The Lens Model thus provides a theoretical framework for distinguishing between how information is gathered and how it is integrated in clinical decision-making.

However, when the results from the two different methods disagree, one cannot follow both of them: When they *disagree*, the final decision will necessarily have to rely on one method over the other [16, 21].

Navigating and integrating different information via the clinical data combination method can be cognitively strenuous and complex [22, 23], especially when the various sources are in conflict with one another. The clinical method has been shown to be less reliable than statistical models [15, 21]. However, the implementation of statistical models can be difficult due to insufficient guidelines, education, or resources, such as a lack of validated advanced statistical models available for practical use in psychological assessments [11]. For example, models that enable the statistical combination of data from several rating scales and clinical history are still uncommon.

Since the introduction of the clinical versus statistical controversy in the 1950s, statistical modeling has undergone significant improvements as a result of advancements in the field of AI [9, 10, 24]. Statistical models, often including AI technology, have become increasingly prevalent in healthcare, especially in medicine [25, 26]. Nevertheless, the question remains whether the benefits of such instruments are used for depression assessments in clinical practice and, if so, to what extent, in what way, and how clinicians’ attitudes reflect their tendencies to use the statistical data combination methods.

### Research support for assessment methods

The validity and reliability of depression assessment methods have been systematically researched for many decades. Semi-structured or structured interviews are often regarded as the benchmark standard for depression assessment because they demonstrate greater validity and reliability than unstructured interviews do [7, 27, 28]. In contrast, unstructured clinical interviews, although widely used in practice, are less reliable and less valid [14, 29], and they are rarely used in research. Self-report questionnaires, while highly reliable, have other shortcomings. For example, rating scales targeting depression: (i) fail to reliably capture the disorder across scales [30], (ii) miss important symptoms [31], (iii) differ substantially in their categorization of depressed patients into severity groups [32], and (iv) are multidimensional, thus multiple constructs with inconsistent factor structures across scales are assessed [33–35].

Across heterogeneous domains, statistical models have demonstrated modest improvements in predictive accuracy over clinical judgment [6, 15, 24, 36]. Despite substantial evidence supporting the reliability and validity of structured assessment methods, as well as evidence suggesting advantages of statistical methods to clinical data combination in some contexts, many clinicians continue to rely primarily on unstructured methods of data collection and clinical judgment as data combination method. This pattern highlights a potential gap between research evidence and clinical practice, although the extent and implications of this gap may vary depending on the specific assessment context.

### Research on depression assessments in practice

With respect to clinical practice, research has examined how mental health professionals use diagnostic classification systems such as the International Classification of Diseases (ICD) [37] or the Diagnostic and Statistical Manual of Mental Disorders (DSM) [38]. Although these systems provide standardized diagnostic criteria, studies suggest that their use in routine practice is variable and often not strictly operationalized [39]. Clinicians may use these frameworks flexibly, combining them with unstructured clinical interviews, personal judgment, and contextual information rather than adhering to fully standardized assessment procedures. Research have further examined what professionals’ attitudes are toward standardized assessment [40–42], and how clinicians’ perceptions of and methods to the assessment process are related [43, 44].

### Study objectives

How these methods are applied in the practices of clinicians is less known, and the available evidence suggests a discrepancy between guidelines on standards of practice and those of clinical practice [8]. This study aims to gain insights into the extent to which standardized assessment methods are used by clinicians, with a particular focus on the assessment of depression, given that depression is highly prevalent and the leading cause of disability globally [45, 46]. Specifically, we investigated (1) collection methods and instruments that clinicians use; (2) the methods used to integrate and interpret these data (i.e., combination methods), including clinical and statistical methods; (3) clinicians’ attitudes toward standardized assessment tools and AI-based decision support; and (4) clinicians’ beliefs about the accuracy of their assessments relative to other methods and other clinicians. These questions were explored across diverse clinical settings, professional roles, and countries, with the goal of understanding the extent to which standardized assessment methods are implemented in real-world clinical contexts and the factors influencing their adoption.

## Methods

### Participants

Clinicians were recruited via convenience- and snowball sampling through several different online platforms such as LinkedIN, Facebook groups, emailing lists, workplace platforms (e.g., Slack channels for clinicians), and Prolific (an online platform where participants can be paid to take part). The inclusion criteria were as follows: clinicians who 1) are currently working or have past experience in clinical practice (physically or digitally) and 2) are assessing or treating depression in patients. Clinicians who had never worked with assessing and/or treating depression were excluded (*n* = 98). The final sample consisted of 585 participating clinicians: 426 (73%) were female, 153 (26%) were male, and six (1%) indicated ‘other’. There were 162 (28%) clinicians from the United Kingdom, 148 (25%) from the United States, 127 (22%) from Sweden, 105 (18%) from the Netherlands, and 43 (7%) from other countries. Among the different occupations reported, psychologists (*n =* 195; 33%) and nurses (*n* = 172; 29%) made up the largest part of the sample. Other occupations were physicians (*n* = 88; 15%), psychotherapists (*n* = 86; 15%), psychiatrists (*n* = 22; 4%), and 78 (13%) of the respondents selected ‘other’ (see Supplementary Table S2 in the Electronic Supplementary Material [ESM] which presents a cross-tabulation of clinician occupations by country). The clinicians reported a mean of 9.08 years (*SD* = 8.2) of experience assessing and/or treating depression. Almost half of the clinicians (*n* = 275; 47%) reported assessing and/or treating depression *daily or several times a week*. The other clinicians reported doing so *once a week or less* (*n* = 118; 20%), *once a month or less* (*n* = 95; 16%), or *having done this previously but not anymore* (*n* = 97; 17%).

### Material: survey content

The survey was designed to gather insights into clinicians’ practices, attitudes, and experiences regarding depression assessment. It included questions on data collection and combination methods, attitudes toward standardized assessment and AI, self-perceived accuracy, clinical experience, and demographics. Rating scales were analyzed as a single category and were not further differentiated by instrument type or psychometric characteristics. The six domains within the questionnaire were (1) *Assessment Methods*, where clinicians reported on the data collection methods they use (e.g., unstructured, semi-structured, and structured interviews; rating scales), frequency of use, and specific instruments for both initial assessment and follow-up; (2) *Data Combination Methods*, where clinicians indicated whether they rely on clinical judgment, statistical models, or single data sources to integrate information; (3) *Assessment Attitudes*, where clinicians were asked about their attitudes regarding the importance of standardized methods for diagnostic assessments, as well as their interest in AI decision support coupled with one of the randomly assigned descriptions (1 = no information on the accuracy of AI, 2 = assuming high accuracy of AI, 3 = empirical data on accuracy of AI); (4) *Self-Perceived Accuracy*, where clinicians compared their accuracy against peers using similar or different methods, including AI and combinations of methods; (5) Clinical *Experience and Confidence*, where clinicians reported their experience with depression assessments, their confidence levels, and time spent per assessment; and (6) *Demographics*, including age, gender, country, profession, therapeutic approach, and employment sector. Detailed descriptions of the questions, response options, and randomized AI descriptions are provided in the ESM, along with the full questionnaire.

### Procedure

Informed consent was obtained from all the clinicians at the start of the survey. They were informed about the nature of the study, that their participation was voluntary and anonymous, that they had the right to withdraw at any time without providing a reason, and that their anonymized answers would be shared according to open science practices. Clinicians completed the survey online, following the question sequence outlined in the Materials section. The clinicians were presented with one of the randomized conditions when asked about their attitudes toward AI decision support. However, owing to an issue with the survey tool, the assigned condition was only recorded for a subset of clinicians, specifically those who completed the survey through Prolific. After completing the survey, the clinicians were debriefed and given the opportunity to send feedback. The survey was available in English, Dutch, and Swedish and took, on average, 10.92 min (SD = 15.89; Med = 7.82) to complete.

### Statistical analyses

The statistical analyses were performed in R (R Core Team, 2022; for details, see ESM). Descriptive statistics (frequencies [Freq], means [*M*], standard deviations [*SD*], medians [Med]) were calculated for each variable. Pearson correlations (*r*) were used to estimate the relationships between the variables, where the cut-off values for the different strengths were as follows: very weak or no correlation (± 0.0-0.2), weak (± 0.3-0.4), moderate (± 0.5-0.6), strong (± 0.7-0.8), and very strong (± 0.9 − 1). Analysis of variance (ANOVA) was employed for the AI condition comparisons and the cross-cultural comparisons. All ANOVAs were calculated for unbalanced designs (Type III ANOVA). The assumptions of homogeneity of variances were checked via Levene’s test and were met. For significant main effects, post-hoc pairwise comparisons were conducted with Bonferroni correction to control for multiple testing. The *alpha*-value was set to 0.05.

To address the nested structure of the data, two supplementary analyses were conducted for the cross-cultural comparisons. First, a linear model with a Country × Profession interaction term was fitted to explicitly test whether profession effects differed across countries. Second, a multilevel model with random intercepts for Country and Profession nested within Country (lmer: Method ~ [1 | Country/Profession]) was fitted to partition variance across levels. These analyses were conducted using the lme4 and lmerTest packages in R [47, 48].

## Results

### Duration of evaluation

The self-reported time it took for clinicians to identify depression in a typical patient ranged from 3 min to 15 hours^1^, with a median time of 60 min (*SD* = 108 min, Mean = 87 min). Detailed distributional properties (including skewness, kurtosis, and quartiles) are provided in Supplementary Table S1 in the ESM. Self-reported assessment time did not correlate significantly with the reported use of various data collection methods (see Figure S3 in the ESM for the correlations between all variables).

### Aim 1: data collection methods

**Fig. 2:**
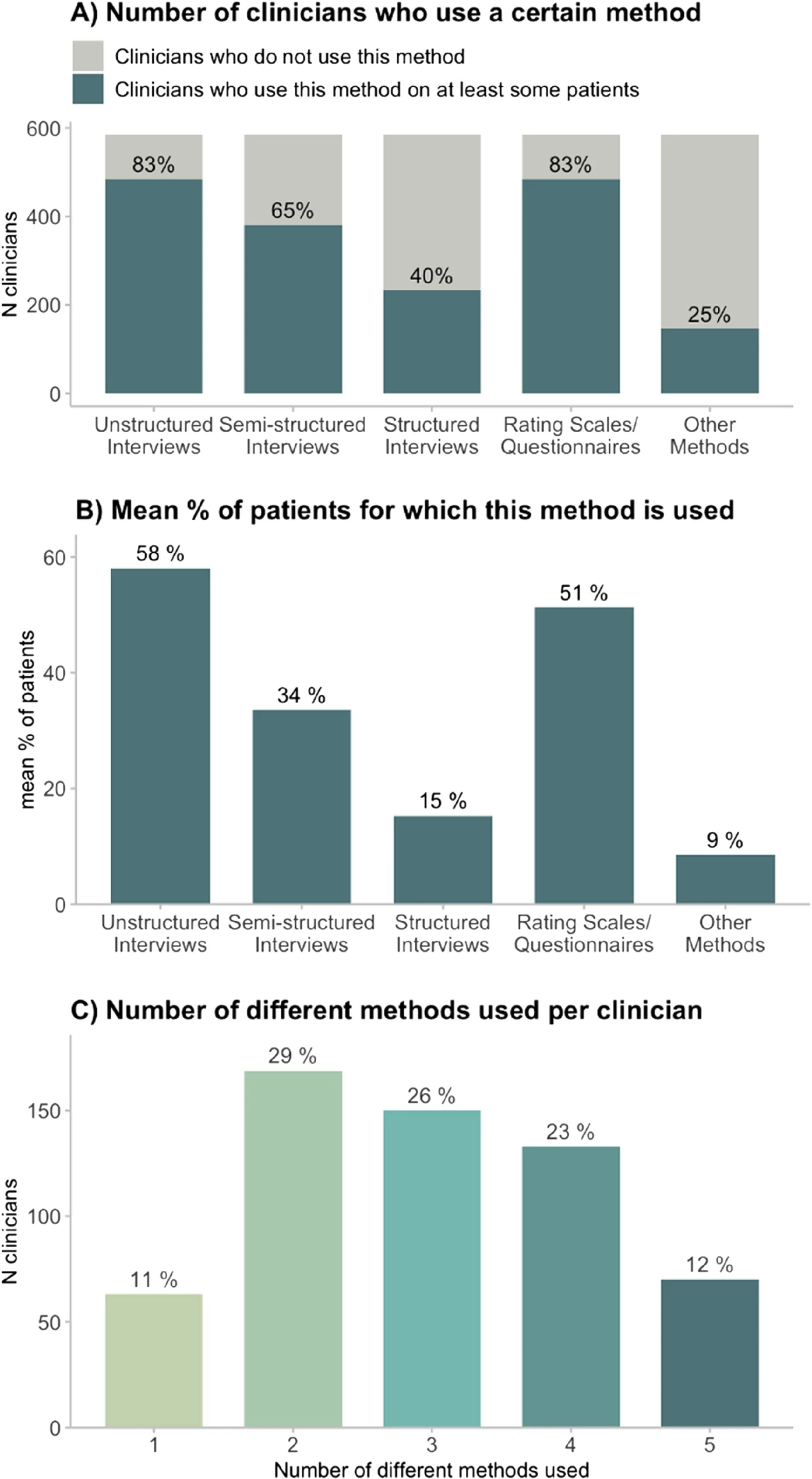
**A**‒**C** Clinicians’ reported data collection methods: **A** number (percentage) of clinicians using each method, **B** mean percentage of patients each method is used for and **C** the number of methods used per clinician across all patients. *N* = 585

The clinicians reported which data collection method(s) they used (Fig. 2a). Most clinicians reported using unstructured interviews (83%) and/or rating scales (83%), followed by semi-structured interviews (65%), structured interviews (40%), and/or ‘other’ methods (25%).

The clinicians also estimated the proportion of their patients for whom they used these different methods (Fig. 2b). On average, they reported using clinical interviews for 58% of their patients, followed by rating scales for 51% of their patients, and semi-structured interviews for 34% of their patients.

Most clinicians (89%) reported using two or more methods across patients (Fig. 2c). Only 11% of the clinicians reported using a single method across all of their patients, of which the unstructured interviews were the most common method. The most commonly reported data collection method combinations were as follows: (1) Unstructured interviews and rating scales (18%); (2) Unstructured interviews, semi-structured interviews, structured interviews, and rating scales (18%); and (3) Unstructured interviews, semi-structured interviews, and rating scales (*n* = 16%).

Most clinicians (84%) indicated performing follow-up assessments; similar patterns regarding the variety of data collection methods were observed among them. A more detailed description is available in the ESM.

#### Specific data collection scales and (semi-)structured interviews

Clinicians could indicate which standardized tools and instruments they use. A list of the specific instruments and their corresponding figures is detailed in the ESM and the *Mental Health Assessment Dashboard* (see MHAD; https://cwiebel.shinyapps.io/ClinicalPractices/), an interactive online tool that allows for further analysis and custom comparisons of the data.

### Aim 2: data combination methods

**Fig. 3:**
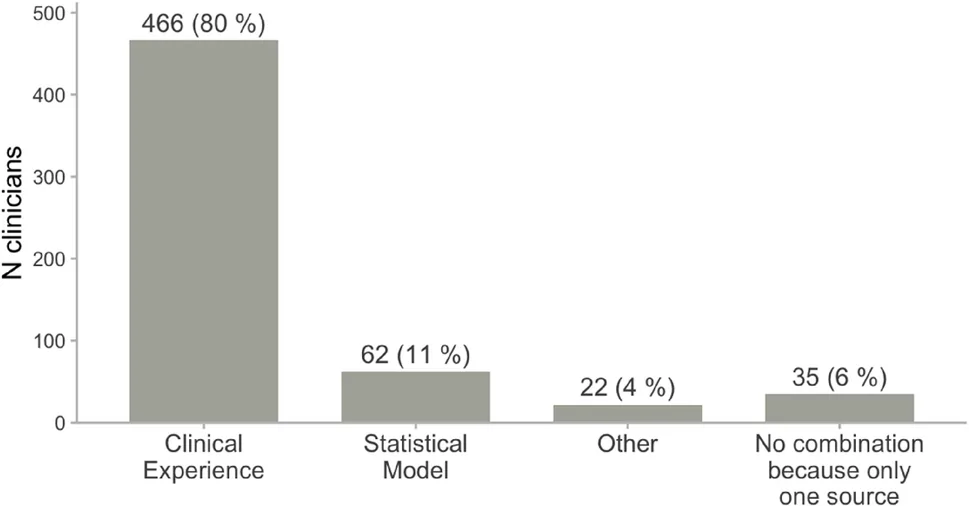
Clinicians’ reported use of different data combination methods. *N* = 585

The vast majority (80%) of the clinicians reported combining the collected data with clinical experience (i.e., the clinical method; Fig. 3). 11% reported using the statistical data combination method, and 6% reported not using any data combination method since they used only one source of information.

### Aim 3: attitudes towards assessment methods

#### Attitudes toward standardized assessment methods

**Fig. 4:**
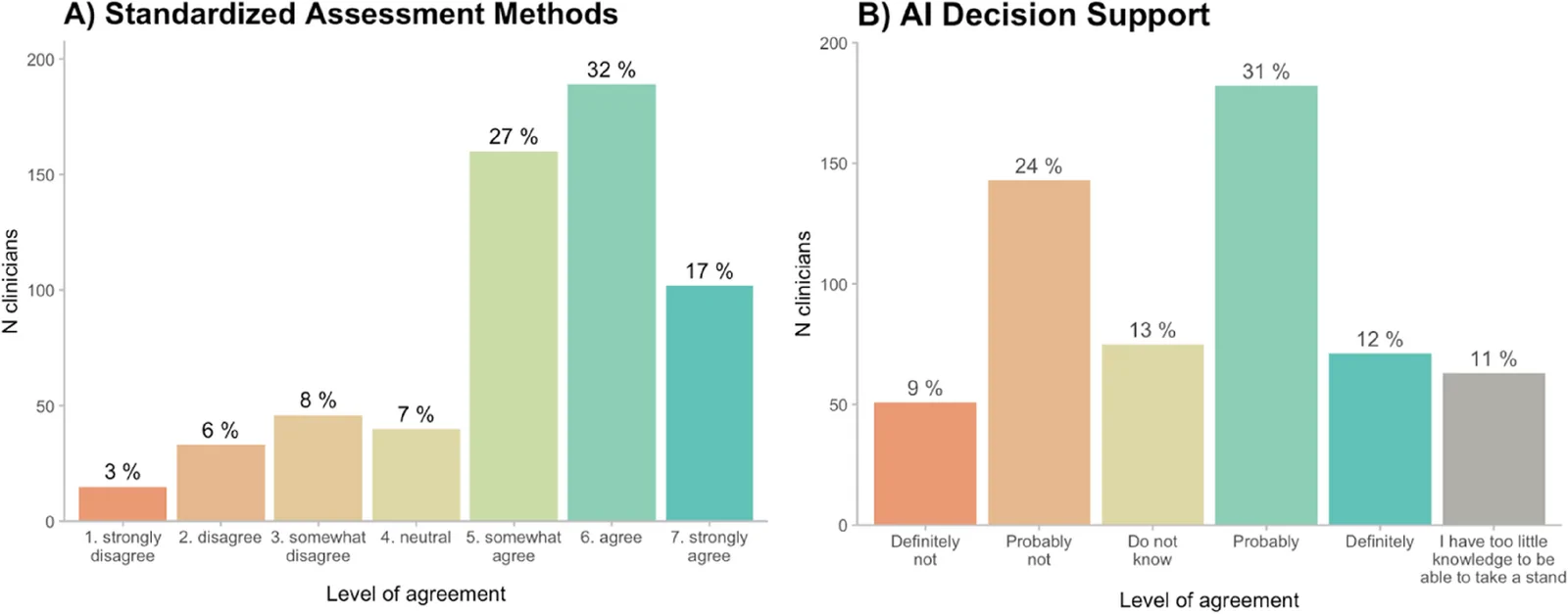
**A**, **B** Clinicians’ reported attitudes toward the use of **A** standardized assessment methods and **B** AI decision support. *N* = 585

More than half of the clinicians reported *somewhat-* to *strongly agree[ing]* (76%) that it is important to use standardized assessment methods with high validity and reliability (Fig. 4a). A notable number of clinicians reported *somewhat-* to *strongly disagree[ing]* (17%) with this statement. Clinicians’ ratings of the importance of using standardized assessments were correlated with their reported frequency of using rating scales (*r* = .20, *p* < .001) and structured interviews (*r* = .14, *p* = .002) but negatively correlated with their reported frequency of using unstructured interviews (*r* = − .13, *p* = .002). Many clinicians have elaborated on their answers. A summary of these elaborations is presented in the ESM.

#### Attitudes toward AI decision support

More clinicians reported to be *probably* or *definitely* interested in receiving AI decision support (43%) than *probably not* or *definitely not* (33%; Fig. 4b). A substantial number reported not knowing or having too little knowledge to take a stand (24%). Greater interest in using AI was correlated with positive agreement on the importance of using standardized assessments (*Spearman’s ρ = 0.116*, *p = .008*) and with the reported frequency of using rating scales (*Spearman’s ρ = 0.164*, *p < .001*). The attitudes toward the use of AI decision support did not significantly differ between the AI information conditions (*F*[2,324] = 1.60, *p* = .204, *n* = 359)^2^. Many clinicians have also elaborated on their answers, which are presented on the ESM.

### Aim 4: beliefs in one’s confidence and accuracy

A majority of the clinicians reported feeling *confident* (35%), *very confident* (32%), or *extremely confident* (10%) in identifying depression, whereas few reported feeling *somewhat confident* (19%), *a little confident* (4%), or *not at all confident* (< 1%). Confidence in identifying depression was positively correlated with the reported assessment frequency of depression (*r* = .33, *p* < .001), work experience with identifying depression (*r* = .21, *p* < .001), and the reported frequency of using rating scales and semi-structured interviews as data collection methods (*r* = .17, *p* < .001 and *r* = .11, *p = .007*). See the ESM for more details.

**Fig. 5:**
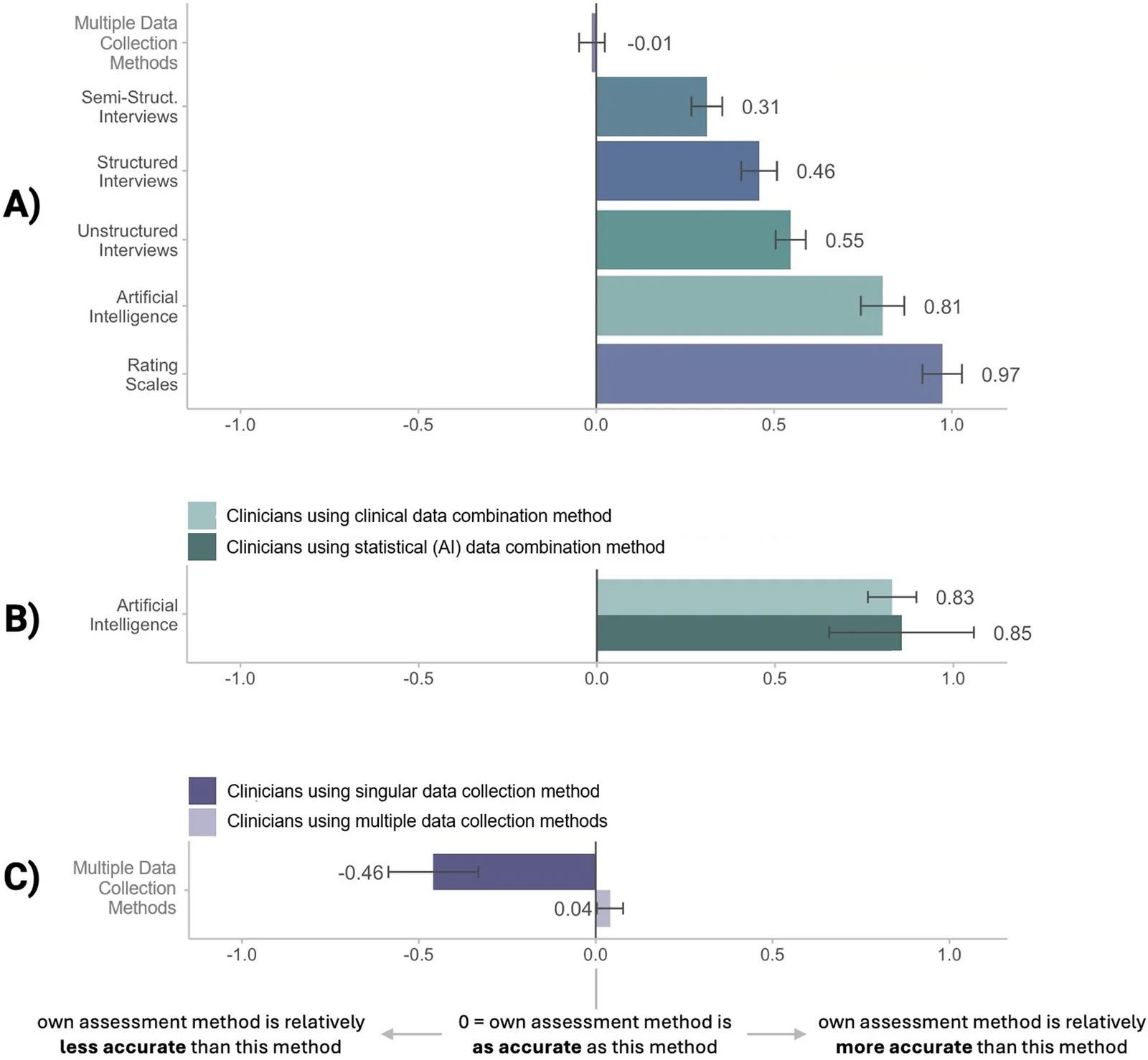
Clinicians' self-perceived assessment accuracy. **A** Overall comparison relative to clinicians using alternative assessment methods. **B** A more detailed analysis of the self-perceived accuracy relative to assessments solely using AI, shown in Panel A, stratified by clinicians' own reported data combination method. **C** A more detailed analysis of the self-perceived accuracy relative to clinicians using multiple data collection methods, shown in Panel A, stratified by clinicians who use a single- versus multiple data collection methods. Error bars represent standard errors of the mean. *N* = 585

Clinicians generally believed that the assessment methods they typically use yielded greater accuracy than methods relying on a single data collection method (Fig. 5A). The largest perceived advantage was observed when comparing their own assessments with those based solely on rating scales. In contrast, clinicians did not perceive their assessments to be more accurate than those of clinicians using multiple data collection methods.

The clinicians who reported relying primarily on the clinical data combination method (80%), perceived their assessments as more accurate than assessments made solely with AI (Fig. 5B). A majority (89%) of the clinicians who reported using multiple data collection methods for their assessments, considered the accuracy of their assessments to be equal to that of other clinicians also using multiple data collection methods (Fig. 5C). Taken together, clinicians generally viewed their own assessment approach as superior to assessments made with a single data collection method and to assessments solely using AI or rating scales, but not compared to the assessment practices of peers who used multiple data collection methods.

### Cross-cultural differences

Exploratory analyses comparing clinicians from the U.S., the Netherlands, Sweden, and the U.K. identified few small to moderate cross-cultural differences. Clinicians from the U.S. and the U.K. used unstructured interviews for the smallest percentage of patients, with averages of 51% and 46%, respectively. The finding that the vast majority of clinicians report combining collected data with clinical experience is consistent across countries. However, these findings should be interpreted with caution due to the heterogeneous nature of the samples, especially with respect to the occupations (e.g., psychologists, psychiatrists, general practitioners, nurses). To address the nested structure of the data, a linear model with an explicit Country × Profession interaction revealed no significant interaction effects (all *p* > .06), and a multilevel model revealed that individual variation accounted for the majority of variance (84.8%), with Country (7.4%) and Profession within Country (7.8%) each explaining modest proportions. For a more exploratory cross-cultural analysis, see the ESM and the *MHAD*.

## Discussion

This study examined the extent to which mental health professionals use standardized assessment methods for assessing depression and their attitudes toward other methods. The results show that depression assessments are conducted by different professions with diverse educational backgrounds, using a wide range of methods and reporting large variations in the time it takes to assess, reflecting real-world practices and the diverse experiences patients encounter. The results show that (1) mental health professionals use a wide variety of data collection methods and that (2) they predominantly rely on the clinical method for data combination. (3) A total of 77% of the clinicians agreed on the importance of using standardized, valid, and reliable assessments, and 43% reported being interested in receiving AI decision support. (4) They are generally confident in their assessments but vary substantially in the reported time it takes to carry out a depression assessment. On average, clinicians believed their own assessments were more accurate than those of AI or other clinicians when based on a single data collection method. Interestingly, a wide variety of methods and practices were consistently found across all the examined countries, with only small significant cross-cultural differences. This highlights the paradox of consistent inconsistency in depression assessment practices on an international scale.

### Data collection methods

Clinicians report using different data collection methods with a wide range of combinations and instruments that vary greatly between individuals. Most clinicians (89%) reported using two or more methods, with unstructured interviews and rating scales being the most common. Although a significant portion of the clinicians (16%) did not conduct follow-up assessments, a similar pattern of diverse methods was observed among those who did. This aligns with previous studies highlighting the lack of use of outcome measures [49, 50]. While unstructured interviews have shown low interrater reliability for diagnosing depression and are seldom used in research [29, 51], various rating scales for assessing depression differ significantly (i.e., they capture different symptoms of depression and only partly overlap [30, 52]; and are often used interchangeably in research [30, 51–53]. This variation in data collection methods may be problematic for providing equal, standardized, high-quality care, especially since research shows that different methods and instruments yield varying results. The absence of a single industry standard for diagnostic manuals further contributes to inconsistencies in assessments and diagnoses [53].

### Data combination methods

While approximately one in ten clinicians reported using the statistical method to form their final assessment, 80% reported interpreting and weighing the collected data on the basis of their clinical experience and judgment. Despite extensive research showing that the statistical method for combining information is as good as or superior to the clinical judgment of an expert clinician [6, 15, 24, 36], the majority of final assessments still rely on the clinical method. As early as 35 years ago, Dawes et al. [16] argued for the routine use of statistical methods in clinical assessment and decision-making. Grove and Meehl [21] addressed this issue, concluding that using the less accurate of two assessment methods “is not only unscientific and irrational, it is unethical” (*p*. 323). However, possible explanations for the continued reliance on clinical data combination might be the lack of easily implemented, user-friendly, and validated statistical models that comply with the required regulations (e.g., CE-marking and FDA approval). Clinical validation and readiness for implementation should be weighed heavily before employing statistical models since, for example, flawed algorithms trained on small or biased training data can have adverse effects [54]. This concern is further amplified by widespread apprehensions about the safety and validity of AI models, as highlighted by clinicians in this survey.

### Attitudes toward standardized assessment

A sizable group of clinicians, approximately one-fifth, reported they *somewhat*- to *strongly disagree* with the importance of using standardized assessment methods that have been demonstrated to have high validity and reliability in research, despite our expectation of near-unanimous agreement. Even though 76% of clinicians reported that they somewhat- to strongly agreed with the statement, the importance they assigned to standardized assessment methods did not appear to influence their final assessment decisions, as most still relied on a combination of clinical data collection methods. This corresponds to other research examining clinicians’ attitudes, which have shown a mostly, but not completely, positive attitude towards standardized assessment methods: Clinicians seem to be positive about the quality of standardized assessment methods but less positive about their usefulness in practice, and the advantages of standardized assessment over clinical judgment [40–42]. To enable better implementation, the need to overcome specific barriers and issues, such as adequate training and practicality, is highlighted [40–42, 44].

### Attitudes toward AI

Clinicians’ interest in AI decision support for depression assessment is mixed: 43% express interest, 33% show disinterest, and 23% remain undecided due to insufficient information. This highlights the need to update clinicians on AI advancements and benefits and involve them in algorithm development and validation. Key concerns, including accountability, transparency, and privacy, must be addressed [55]. Interest in AI support correlates with the use of rating scales and positive attitudes toward standardized methods, suggesting that clinicians familiar with these practices could facilitate AI integration in clinical settings. However, enhanced education on AI and statistical models is essential. For example, a study on psychiatrists’ views of generative AI found widespread use for administrative tasks but skepticism toward its active role in patient care [56]. The lack of differences in attitudes across AI descriptions and diverse responses in open comments further emphasize the need for comprehensive educational initiatives to foster an understanding of AI’s capabilities, limitations, and ethical integration into mental health practices.

### Clinicians assessment accuracy

Strikingly, a majority of clinicians who rely on a combination of clinical data believe that their assessments are more accurate than those derived from a statistical method or AI, which contradicts findings from previous research [6, 15, 24, 36]. The clinicians ranked the accuracy of their assessments as superior to those conducted by peers who use only a single method (particularly rating scales alone) but not compared with those using a combination of methods. These findings suggest a general belief among clinicians in the superiority of employing multiple methods for data collection and combining data via a clinical method. The belief in the clinical method could be explained by a more general tendency of individuals to overestimate their cognitive abilities [57]. However, clinicians’ confidence and accuracy beliefs have been shown to be poor indicators of their assessment accuracy [20, 23, 58], which is troubling given the impact it may have on patients and their course of treatment.

## Limitations

Despite the prevalence and clinical significance of depression, research on its clinical assessment methods remains limited. The present study begins to address this gap, yet certain limitations constrain the results and highlight important directions for future research to further our understanding of depression assessment practices and facilitate the adoption of standardized assessment methods. Given the study’s broad focus on depression assessments in real-world clinical settings, the sample comprises a wide range of occupations, including psychologists, psychotherapists, psychiatrists, and general practitioners. However, this makes it challenging to understand the experiences of specific occupations; therefore, future research could focus on single professional groups to gain deeper insights into their unique practices and challenges. Similarly, the goals and methods of clinicians’ assessments can differ significantly both between and within countries, occupations, and clinics, all of which warrant further investigation. The data have a nested structure (e.g., clinicians clustered within profession–country groups), which is not fully accounted for in the current analyses. This may affect standard error estimates and limits interpretation of group-level effects. Future research should consider multilevel modeling approaches. Although clinicians reported using multiple assessment methods, the survey design did not allow for the construction of standardized “assessment batteries” or composite profiles reflecting typical combinations of tools across patients. As a result, we were unable to identify common or “preferred” assessment configurations. Future research could explicitly model such combinations to better understand typical assessment pathways and their variability across clinicians and contexts. The present study did not include detailed measures of clinicians' workload, job duties, or task-specific constraints, which may influence the selection and application of assessment methods in practice. Similarly, although clinicians reported confidence in their assessments, the study did not include objective measures of competence or calibration between confidence and accuracy. Prior research has shown that self-reported competence can incrementally predict intentions to use assessment methods beyond other training-related factors [59], suggesting that perceived competence plays a meaningful role in shaping assessment practices. Future research should examine how contextual factors such as workload and job responsibilities interact with assessment practices, and how self-perceived competence relates to decision-making processes and method selection among practicing clinicians. The study did not include symptom-level data, detailed information on how individual symptoms were assessed, or how clinicians structured follow-up decisions (e.g., timing, intensity, or escalation of assessment). These elements represent important components of clinical decision-making and may influence both assessment accuracy and method selection. Future studies should incorporate more granular process-level data to better capture how clinicians evaluate and act upon specific symptom presentations over time. This study included only Western countries, each with distinct guidelines, rules, and regulations governing mental health practices. The distribution of occupations also varies across countries, which necessitates caution when interpreting cross-country differences. Future research should expand to include non-Western countries to gain insights into how mental health practices are carried out in diverse cultural and regulatory contexts.

Additionally, this study specifically examined the assessment of depression, so the findings may not be generalizable to other mental health conditions. The use of a self-report survey format introduces the potential for recall bias and may reflect clinicians’ perceptions of best practices rather than their actual behaviors. As a result, the data could overestimate the extent to which standardized assessment methods are employed in practice. The study did not differentiate between types of self-report instruments with respect to their psychometric properties or level of standardization (e.g., norm-referenced instruments with embedded validity indicators versus brief symptom screeners). As a result, the category of rating scales includes heterogeneous instruments that may differ substantially in validity, reliability, and clinical function. Future research should distinguish between these types of instruments and examine whether clinicians’ choice of instrument is informed by awareness of its psychometric properties, to better understand how measurement quality and accessibility influence assessment practices across contexts and countries. The analyses examining associations between attitudes toward AI and other variables relied primarily on correlation-based approaches. Given the distributional properties of some variables (e.g., skewed responses and categorical or near-binary attitude measures), these analyses should be interpreted with caution. An additional avenue for future research is to examine how clinicians’ attitudes toward AI in assessment align with emerging ethical frameworks in professional psychology, including cross-cultural differences in how such ethical considerations are interpreted and applied.

Future studies could apply more refined analytical approaches (e.g., non-parametric methods or distribution-sensitive modeling) to better capture relationships between attitudes and assessment practices. In addition, AI-related randomization data were missing for some participants, which reduced the statistical power. Finally, online recruitment and sampling may have restricted participation to specific subgroups. The sampling strategy is also likely to favor digitally active clinicians, which should be considered when interpreting the findings. Future research could benefit from random sampling directly from clinical settings or from observational studies of audits of actual practices, etc.

### Potential implications and future research

A better understanding of current depression assessment practices can provide clinicians, policymakers, and researchers with a foundation for improving healthcare quality. Understanding current practices can support clinicians, regulatory bodies, and researchers in joining forces to lead changes and update guidelines and policies. The Mental Health Assessment Dashboard can inform discussions on the current (*limited*) use of standardized assessment methods and any future integration of AI in clinical settings. Furthermore, there is a need for more research regarding the potential risks of current depression assessment practices and their implications for patients and the challenges for clinicians in embracing newer standardized assessment methods [17, 60]. There is a lack of research on how clinicians combine assessment methods and the potential shortcomings associated with the clinical combination method.

The variability in assessors and assessment methods reflects real-world practice and highlights the need for clearer guidelines and training to ensure equitable and consistent standardization. In other words, the outcome of an assessment for depression should not depend on where, when, or by whom the patient is assessed. Additionally, many clinicians’ concerns about AI should be addressed, including biases and ethical issues, as well as how they can work with AI. Transparent communication and education about the capabilities and limitations of AI, along with evidence from robust studies, can potentially build trust and acceptance among clinicians [61].

## Conclusion

Previous research [7, 53] has typically studied depression assessment methods in research settings or focused on attitudes in clinical settings [40–42] rather than actual assessment practices in different clinical practices. Our results highlight heterogeneity in depression assessments: Many different professionals conduct depression assessments in clinical practice (e.g., psychiatrists, psychologists, general practitioners, nurses); employ a wide variety of methods (ranging from unstructured to structured methods using numerous tools) in different combinations, and report a great difference in the time it takes to carry out the assessment.

The results reveal a pattern of skepticism toward AI, underestimation of statistical models, and overestimation of one’s own accuracy, which aligns with findings from previous studies. While clinicians express confidence in their ability to assess depression, the widespread use of unstructured interviews alongside standardized tools, coupled with a predominant reliance on clinical judgment over statistical models, suggests a disconnect between best practices supported by research and everyday clinical practice. Adopting more standardized methods in the assessment processes, be it during the data collection and/or during the combination of data, is essential to synthesize information systematically and improve the quality of the data used in clinical judgment. This alignment between clinical practice and research evidence would enhance patient care while allowing clinicians to consider individual factors. However, achieving this requires harmonizing clinical judgment with data-driven methods, as well as aligning manuals and guidelines with empirical research. Future initiatives should focus on developing accessible, practical, validated tools and training programs that bridge the gap between research and practice, fostering integration and consistency in depression assessment.

## Supplementary Information

Supplementary Material.

## Data Availability

The datasets generated and/or analysed during the current study are available at [https://osf.io/pw8mk/]. The code for the *Mental Health Assessment Dashboard* is also shared here in case the app no longer can be hosted [https://cwiebel.shinyapps.io/ClinicalPractices/]. Open (anonymized) data, code, and surveys are available at https://osf.io/pw8mk/; including an open updated report, reflecting the most current data collected post publication and changes over time.

## Footnotes

1 Nine cases with a reported time greater than the mean plus 3 standard deviations were seen as outliers and thus removed from the time analysis. Their reported assessment times ranged from 20 to 60 h, respectively. Additionally, seven cases reported 0 min; these were removed because it is possible that they do not assess depression at all.

2 The lower number of clinicians in this analysis is due to the randomisation condition not being saved for all clinicians.

## Abbreviations

AI: Artificial Intelligence
DSM: Diagnostic and Statistical Manual of Mental Disorders
ESM: Electronic Supplementary Material
ICD: International Classification of Diseases
LLM: Large Language Model (only include if mentioned earlier; I didn’t sit explicitly)
MHAD: Mental Health Assessment Dashboard
U.K.: United Kingdom
U.S.: United States
WHO: World Health Organization
