# Supplementary material for: Clinicians’ practices and attitudes toward depression assessment: a cross-sectional survey study
Source: BMC Psychol. 2026 Aug 19;14:1227. doi: 10.1186/s40359-026-05036-w (PMC13491592; doi:10.1186/s40359-026-05036-w)
Supplement: Supplementary file 1 [file 40359_2026_5036_MOESM1_ESM.docx]

**Supplementary Material:**

**Clinicians’ Practices and Attitudes Toward Depression Assessment: A Cross-Sectional Survey Study**

[**Elaboration on Ethics**	2](#_Toc236058714)

[**Participants**	2](#_Toc236058715)

[**Descriptive Statistics**	2](#_Toc236058716)

[**Elaborations of Attitudes**	4](#_Toc236058717)

[**Attitudes Toward Standardized Assessment Methods**	4](#_Toc236058718)

[**Summary of Responses**	4](#_Toc236058719)

[Agreement	4](#_Toc236058720)

[Disagreement	5](#_Toc236058721)

[Mixed agreement/disagreement	5](#_Toc236058722)

[**Attitude Towards AI Decision-Support**	5](#_Toc236058723)

[Agreement	5](#_Toc236058724)

[Disagreement	6](#_Toc236058725)

[Mixed agreement/disagreement	6](#_Toc236058726)

[**Elaboration of** **Cultural Comparisons**	6](#_Toc236058727)

[**Assessments**	7](#_Toc236058728)

[Duration of assessment	7](#_Toc236058729)

[Data collection methods across countries	8](#_Toc236058730)

[Data combination methods across countries	12](#_Toc236058731)

[Attitudes toward standardized assessment methods and AI across countries	12](#_Toc236058732)

[**Pearson Correlations**	14](#_Toc236058733)

[**Follow up**	15](#_Toc236058734)

[**Survey Questionnaire**	16](#_Toc236058735)

[**References**	21](#_Toc236058736)

## **Elaboration on Ethics**

According to Swedish law (2003:460) and the Swedish Ethical Review Authority, research must undergo external ethical review whenever it involves i) collecting personal information, ii) presenting an “obvious” risk for physical or psychological harm, or iii) manipulating or deceiving individuals. Since this research involves asking clinicians about their assessment method at work, it does not fall within these criteria.

## **Participants**

Recruitment was made on several different online platforms such including LinkedIN, Facebook groups, emailing lists, workplace platforms (e.g., Slack channels for clinicians), and Prolific (an online platform where participants can be paid to take part)

## **Descriptive Statistics**

**Table S1**

*Descriptive Statistics for Key Variables by Country*

| **Variable** | **Country** | **N** | **M** | **SD** | **Mdn** | **Q1** | **Q3** | **Min** | **Max** | **Skew** | **Kurtosis** |
| --- | --- | --- | --- | --- | --- | --- | --- | --- | --- | --- | --- |
| ***Experience (years)*** | Other | 43 | 6.36 | 8.14 | 3.08 | 1.62 | 7.79 | 0.08 | 39.17 | 2.22 | 4.9 |
|  | Sweden | 127 | 12.99 | 8.79 | 10.33 | 7 | 17.58 | 0.08 | 43 | 1.16 | 1.39 |
|  | The Netherlands | 105 | 8.58 | 7.53 | 6.25 | 3.5 | 12.17 | 0 | 38 | 1.61 | 2.96 |
|  | United Kingdom | 162 | 8.17 | 8.01 | 5 | 2.6 | 10.92 | 0.25 | 41 | 1.69 | 2.79 |
|  | United States | 148 | 7.85 | 7.34 | 5.33 | 3 | 10 | 0.17 | 40 | 1.75 | 3.27 |
| ***Assessment time (hours)*** | Other | 43 | 1.77 | 3.16 | 1 | 0.67 | 1.5 | 0 | 20 | 4.64 | 23.24 |
|  | Sweden | 127 | 1.72 | 5.36 | 1 | 0.5 | 1.5 | 0 | 60 | 10.17 | 107.43 |
|  | The Netherlands | 105 | 2.12 | 4.91 | 1.5 | 1 | 2 | 0.08 | 50 | 8.86 | 83.07 |
|  | United Kingdom | 162 | 2.07 | 5.87 | 1 | 0.5 | 2 | 0 | 60 | 7.66 | 65.14 |
|  | United States | 148 | 2.46 | 7.03 | 0.79 | 0.25 | 1.5 | 0 | 60 | 5.88 | 38.49 |
| ***Confidence*** | Other | 43 | 3.95 | 1.15 | 4 | 3 | 5 | 2 | 6 | 0 | -0.86 |
|  | Sweden | 127 | 4.36 | 0.88 | 4 | 4 | 5 | 2 | 6 | -0.35 | -0.07 |
|  | The Netherlands | 105 | 4.1 | 0.81 | 4 | 3 | 5 | 3 | 6 | 0.04 | -1.07 |
|  | United Kingdom | 162 | 4.07 | 1.1 | 4 | 3 | 5 | 1 | 6 | -0.13 | -0.53 |
|  | United States | 148 | 4.47 | 1.03 | 4.5 | 4 | 5 | 2 | 6 | -0.22 | -0.62 |
| ***Interview time (sec)*** | Other | 43 | 900.38 | 2460.57 | 443.05 | 354.7 | 663.16 | 180.39 | 16538.1 | 5.96 | 34.94 |
|  | Sweden | 127 | 774.57 | 767.19 | 603.6 | 448.31 | 895.66 | 234.72 | 8361.86 | 7.7 | 72.48 |
|  | The Netherlands | 105 | 893.46 | 1195.1 | 610.43 | 427.13 | 821.99 | 202.7 | 9754.6 | 5.04 | 29.88 |
|  | United Kingdom | 162 | 460.57 | 302.23 | 368.55 | 300.99 | 524.75 | 159.34 | 2788.33 | 3.76 | 22 |
|  | United States | 148 | 525.82 | 373.16 | 423.76 | 316.73 | 575.72 | 160.07 | 2684.37 | 3.15 | 12.66 |
| ***Unstructured interview (%)*** | Other | 43 | 46.63 | 38 | 35 | 20 | 85 | 0 | 100 | 0.23 | -1.52 |
|  | Sweden | 127 | 65.27 | 41.93 | 90 | 20 | 100 | 0 | 100 | -0.64 | -1.38 |
|  | The Netherlands | 105 | 78.8 | 33.34 | 100 | 60 | 100 | 0 | 100 | -1.36 | 0.38 |
|  | United Kingdom | 162 | 48.69 | 37.01 | 50 | 20 | 80 | 0 | 100 | 0.1 | -1.47 |
|  | United States | 148 | 50.54 | 38.03 | 50 | 15 | 90 | 0 | 100 | 0.03 | -1.53 |
| ***Semi-structured interview (%)*** | Other | 43 | 39.56 | 35.13 | 30 | 9.5 | 65 | 0 | 100 | 0.46 | -1.21 |
|  | Sweden | 127 | 28.54 | 37.91 | 0 | 0 | 50 | 0 | 100 | 0.96 | -0.67 |
|  | The Netherlands | 105 | 32.58 | 38.03 | 10 | 0 | 75 | 0 | 100 | 0.7 | -1.15 |
|  | United Kingdom | 162 | 34.54 | 33.62 | 25 | 0 | 50 | 0 | 100 | 0.77 | -0.62 |
|  | United States | 148 | 35.53 | 35.64 | 20 | 0 | 52.5 | 0 | 100 | 0.68 | -1 |
| ***Structured interview (%)*** | Other | 43 | 22.44 | 29.41 | 10 | 0 | 35 | 0 | 100 | 1.22 | 0.39 |
|  | Sweden | 127 | 13.78 | 29.87 | 0 | 0 | 10 | 0 | 100 | 2.12 | 2.94 |
|  | The Netherlands | 105 | 17.57 | 32.26 | 0 | 0 | 20 | 0 | 100 | 1.77 | 1.6 |
|  | United Kingdom | 162 | 14.65 | 25.35 | 0 | 0 | 20 | 0 | 100 | 2.07 | 3.7 |
|  | United States | 148 | 13.49 | 24.8 | 0 | 0 | 15 | 0 | 100 | 2.27 | 4.53 |
| ***Rating scales (%)*** | Other | 43 | 40.49 | 39.62 | 25 | 2.5 | 85 | 0 | 100 | 0.5 | -1.44 |
|  | Sweden | 127 | 65.71 | 38.45 | 80 | 25 | 100 | 0 | 100 | -0.58 | -1.34 |
|  | The Netherlands | 105 | 54.59 | 39.9 | 50 | 20 | 100 | 0 | 100 | -0.08 | -1.69 |
|  | United Kingdom | 162 | 42.32 | 38.94 | 30 | 10 | 90 | 0 | 100 | 0.44 | -1.42 |
|  | United States | 148 | 49.39 | 40.4 | 50 | 10 | 100 | 0 | 100 | 0.11 | -1.68 |
| ***Other methods (%)*** | Other | 43 | 14.19 | 24.88 | 0 | 0 | 17.5 | 0 | 100 | 2.15 | 4.16 |
|  | Sweden | 127 | 9.33 | 24.66 | 0 | 0 | 0 | 0 | 100 | 2.9 | 7.41 |
|  | The Netherlands | 105 | 12.77 | 26.37 | 0 | 0 | 10 | 0 | 100 | 2.23 | 4.04 |
|  | United Kingdom | 162 | 5.2 | 15.55 | 0 | 0 | 0 | 0 | 100 | 4.37 | 21.09 |
|  | United States | 148 | 6.79 | 18.81 | 0 | 0 | 0 | 0 | 100 | 3.57 | 13.03 |
| ***Age (years)*** | Other | 43 | 34.44 | 11.73 | 29 | 25.5 | 40 | 22 | 62 | 1 | -0.35 |
|  | Sweden | 127 | 45.42 | 11.46 | 44 | 37 | 54 | 10 | 73 | 0.17 | -0.1 |
|  | The Netherlands | 105 | 36.57 | 9.56 | 36 | 29 | 41 | 19 | 64 | 0.79 | 0.09 |
|  | United Kingdom | 162 | 38.27 | 11.83 | 36.5 | 29 | 44 | 5 | 69 | 0.63 | 0.1 |
|  | United States | 148 | 37.01 | 11.22 | 34 | 29 | 41 | 21 | 76 | 1.19 | 1.2 |

Note. N = Number of participants; M = mean; SD = standard deviation; Mdn = median; Q1/Q3 = first/third quartile. Assessment time and interview time variables show high skewness due to extreme outliers. Percentages reflect the proportion of patients for whom each method is used.

## **Elaborations of Attitudes**

### **Attitudes Toward Standardized Assessment Methods**

Many clinicians (*n* = 237) elaborated on their level of agreement with the statement: “When assessing depression, it is important to use standardized assessment methods…”. Three authors structured these answers into common themes (C.W., D.N., V.E.; see open material for all anonymized, open answers).

### **Summary of Responses**

#### Agreement

Some clinicians emphasized the importance and effectiveness of using standardized methods to minimize human errors and biases. They noted: “We are human and mental health is a complex phenomenon, which is why standardized and reliable tools are needed.”

Others argued for using a combination of unstructured interviews and standardized methods to complement each other. One example of a statement is: “It is important because you cannot rely solely on clinical experience and conversations/interviews, you can miss something. [...] ”.

#### Disagreement

Many also expressed concerns about using standardized assessment methods. Two of the most commonly reported arguments against standardized methods were that they do not cover all aspects of a patient clinical picture. One such response is: “I think that qualitative information can provide a much richer detail of the person's experiences. [...]”. Another response affirms how unstructured interviews are sufficiently valid, suggesting that “[...] The clinical unstructured interview is often sufficient. Diagnosing depression is not that complicated.”

#### Mixed agreement/disagreement

Many also reported that they would rather focus on individualized treatment and the therapeutic relationship with the patient than on obtaining the most accurate assessment since it would have little relevance to the choice of treatment or the quality of care provided, writing, “I assess my patients for the treatment I provide. Whether or not the patients meet the diagnostic criteria is rarely relevant to the choice of method [...]”. Some clinicians reported practical issues mainly concerning time constraints: “It takes an awful lot of time to classify according to the rules of the DSM (with accompanying questionnaires and interviews), which takes away from the time and attention for the emergence, maintenance, protective factors, the building of treatment relationships, etc. [...]”.

### **Attitude Towards AI Decision-Support**

Many clinicians (*n* = 211) elaborated on their answers regarding AI-decision support, which three of the co-authors (C.W., D.N., V.E.) structured into common themes (see open material for all anonymized, open answers).

#### Agreement

Many stated an interest in implementing AI decision support in assessing depression, as long as it could be incorporated well into the clinical practice and provides positive value, one such comment read, “[...] As long as this can be deployed safely, well (scientifically) substantiated and according to privacy rules, I am open to this, as long as it benefits the patient.”. Others highlighted the benefits of AI over human capacities, noting, “I am positive about using AI to support assessment and treatment because algorithms can pick up on things that a human is not able to detect [...].”

#### Disagreement

Issues regarding potential biases, privacy, accountability, or the risk-to-benefit ratio were recurring topics among the comments, with some examples of those being “ [...] I am concerned about how to detect bias in AI models.”; “Privacy is an important issue for me in this”, “[...] I think there is a danger that a therapist could start to ‘hide’ behind the AI information, that it could lead to tunnel vision [...].”; and “I have a very difficult time seeing how this would be additive in any way while the risks are clear and significant.”.

The capability of AI compared to the superiority of clinical experience and human judgment was one aspect that was recurring, with one such example reading, “I trust human judgment more than a human-made algorithm run through a data set. You probably aren’t going to follow the outcome that would roll out anyway, even or especially if that outcome deviates enormously from your clinical judgment. [...]”.

#### Mixed agreement/disagreement

Many also suggested that a person seeking treatment wants to be seen as a human being, which an AI cannot provide, writing, “It sounds interesting, but I would be concerned about this being impersonal and having the potential to miss the human being underneath the difficulties.” The most frequently expressed reservation was that there is a lack of research regarding the assessment accuracy of AI instruments and the need for more information on the AI in question. One respondent wondered: “[...] what is the information based on, how was it generated/using what information and algorithms [...]”.

## **Elaboration of** **Cultural Comparisons**

The cross-cultural analyses compare the Netherlands, Sweden, the U.S., and the U.K. Table S2 illustrates the distribution of occupations within each country, showing significant imbalance between the different represented occupations (e.g., Psychologist, Psychotherapist, General Practitioner, Nurses) within each country. While occupational diversity highlights the extensive range within the mental health assessment field, it hinders our cross-cultural comparisons due to the numerous comparison groups with significant sample-size imbalances, including several with very few cases. The majority of clinicians from the Netherlands and Sweden were Psychologists or Psychotherapists, whereas in the U.K., the majority were Nurses. Since the occupation of the respondents might confound the findings of the cross-cultural comparisons, we decided to include occupation as a second variable in the ANOVAs. All ANOVAs were calculated for unbalanced designs (type “III” ANOVA) and did not assess interaction effects due to the dependence between occupation and country.

The occupation categories “psychiatrist” and “occupational therapist” were removed from the cross-cultural analyses because there were too few in these categories. Moreover, the “other” categories within the country and occupation were removed because they were too heterogeneous. For the cross-cultural analyses, the occupation variables were recoded so that each clinician only had one job title; the occupation title requiring the highest education in Psychology was chosen for analysis. The exclusions resulted in a sample of *n* = 476 for the exploratory cross-country comparisons. In the survey, clinicians could indicate more than one occupation.

For more detailed comparisons, e.g., across age groups, or a combination of variables, we invite the reader to explore the *Mental Health Assessment Dashboard* and the open data (<https://cwiebel.shinyapps.io/ClinicalPractices/>).

| **Table S2 \| Cross-tabulation table for the occupations within each country.** | | | | |
| --- | --- | --- | --- | --- |
| **Psychotherapist** | 39 | 18 | 21 | 6 |
| **Psychologist** | 54 | 81 | 25 | 9 |
| **Physician / GP** | 3 | 14 | 27 | 25 |
| **Occupations** | **The Netherlands** | **Sweden** | **The U.S.** | **The U.K.** |
| **Nurse** | 2 | 2 | 61 | 89 |
| **Notes.** GP = General Practitioner | | | | |

### **Assessments**

#### Duration of assessment

Reported assessment times for depression varied widely across clinicians (Figure S1A); but did not significantly differ by country (main effect for country: *F*(3, 469) = .486, *p* = .692) nor occupations (main effect: *F*(3, 469) = .431, *p* = .233; see Figure S1B).

**Figure S1 A-B | Self-reported Time Taken Identifying Depression: A) Distributions of hours taken to identify depression and B) Comparisons across countries.**
Question: *On average, approximately how much time do you spend on identifying depression for a typical patient showing signs of depression or low mood? n* = 476

#### Data collection methods across countries

Figure S2 presents the data collection methods used by clinicians across the four countries. The differences across the countries (i.e., the effects of country) are described in the main paper. Regarding occupation, there was a small but significant effect on the use of unstructured interviews (main effect: *F*(3, 469) = 4.76, *p* = .003, η² = .03). After Bonferroni correction, the main effect of occupation was only significant for the use of rating scales (*F*(3, 469) = 15.02, *p* < .001).

**Percentage of clinicians who use a certain method**

A) **Mean percentage of patients for which this method is used**

B) **Number of different methods used per clinician**

**Figure S2 A-C | Data Collection Methods Across Countries: A) Percentage of clinicians using each method, B) Mean percentage of patients each method is used for, and C) Number of methods used per clinician (although not necessarily for each patient).**
Question: *What method(s) do you use, and how often do you use them when identifying, assessing, and/or excluding (ruling out) depression?* n = 476.

Figure S3 shows specific questionnaires and (semi-)structured interviews clinicians use for the different data collection methods across the four countries. Main findings tend to hold across the four countries – We only find a few substantial exceptions, such as the PHQ-9 being the most frequently used rating scale in all countries except for the Netherlands, where they most frequently report using the BDI, and Sweden, where MADRS is preferred.

Among the clinicians who indicated which structured or semi-structured interviews they use, the DIS is more commonly reported in the U.K. and the U.S. (although the MINI is also reported being used); and the SCID is the most commonly reported structured interview across all four countries.

**Figure S3 A-D | Clinicians’ reported use of data collection instruments for assessing depression across rating scales, semi-structured interviews, and structured interviews.**

Questions: *Which rating scale(s) [semi-structured interview(s) / structured interview(s)] do you use for assessing depression?*

*BDI = Beck Depression Inventory, CBCL = Child Behavior Checklist, CDI = Children's Depression Inventory, CDRS = Children’s Depression Rating Scale, CES = Center for Epidemiologic Studies Depression Scale, DASS = Depression and Anxiety Stress Scales, GDS = Geriatric Depression Scale, HDRS = Hamilton Depression Rating Scale, IDS = Inventory of Depressive Symptomatology, MADRS = Montgomery-Åsberg Depression Rating Scale, PHQ = Patient Health Questionnaire, QIDS = Quick Inventory of Depressive Symptoms, ADIS = Anxiety and Related Disorders Interview Schedule for DSM-5, DIAMOND = Diagnostic Interview For Anxiety, Mood, And Obsessive-Compulsive And Related Neuropsychiatric Disorders, KSADS = Kiddie Schedule for Affective Disorders and Schizophrenia, SADS = Schedule for Affective Disorders and Schizophrenia, SCAN = Schedules for Clinical Assessment in Neuropsychiatry, SCID = The Structured Clinical Interview for DSM-5, CIDI = Composite International Diagnostic Interview, DIS = Diagnostic Interview Schedule, MINI = Mini International Neuropsychiatric Interview*

#### Data combination methods across countries

Figure S4 shows specific data combination methods clinicians use across the four countries. The differences across the countries (i.e., the effects of the country) are detailed in the main paper.

**Figure S4 | Clinicians’ reported use of different data combination methods across countries**

Question: *How do you typically weigh the gathered information from different sources when assessing depression? n* = 476.

#### Attitudes toward standardized assessment methods and AI across countries

The reported attitude towards standardized methods differs across countries (main effect: *F*(3, 469) = 7.35, *p* < .0001, η² = .04), but not occupations (main effect: *F*(3, 469) = 0.59, *p* = .621). The largest difference was noted between the U.S. (M = 5.64) and the U.K. (M = 4.88), even though the overall differences were relatively small.

The interest in receiving AI decision support also differed significantly across countries (main effect: *F*(3, 469) = 10.56, *p* < .001, η² = .07; Figure S5), but not occupations (main effect: *F*(3, 469) = 0.88, *p* = .604), where Sweden (M = 3.67) demonstrated the highest interest, and the U.K. (M = 2.76) the lowest interest. Across the four countries, the effect size is medium (Cohen, 1988).

**Figure S5 A-B | Clinicians' attitudes towards using A) standardized assessment methods and B) AI-decision support across countries.**
Questions: a) When assessing depression, it is important to use standardized assessment methods that are demonstrated in research to have high validity^1^ and reliability^2^ (^1^The degree to which the assessment method measures what it is supposed to measure; ^2^The degree to which the assessment method produces similar results under similar conditions); and b) If given the opportunity, would you be interested in receiving decision support from Artificial Intelligence (AI) when assessing depression? n = 476.

| **Table S3 \|** Cross-tabulation of clinicians' attitudes toward standardized assessment methods and interest in AI decision support. | | | |
| --- | --- | --- | --- |
|  | **Interested in AI** | **Neutral on AI** | **Not Interested in AI** |
| **Endorses standardized methods** | 51.2% | 13.9% | 34.8% |
| **Neutral to Standardized Assessment View** | 41.2% | 14.7% | 44.1% |
| **Opposes standardized methods** | 38.4% | 16.3% | 45.3% |
| Note. Percentages represent the proportion of clinicians within each standardized assessment attitude category who reported interest, neutrality, or disinterest in AI decision support. *N* = 585. | | | |

## **Pearson Correlations**

| **Table S4 \| Pearson Correlations.** | | | | | | | | | | | |
| --- | --- | --- | --- | --- | --- | --- | --- | --- | --- | --- | --- |
| **Variables** | **1** | **2** | **3** | **4** | **5** | **6** | **7** | **8** | **9** | **10** | **11** |
| **1. Confidence** | - |  |  |  |  |  |  |  |  |  |  |
| **2. Experience (years)** | .21*** | - |  |  |  |  |  |  |  |  |  |
| **3. Assessment frequency** | .33*** | .06 | - |  |  |  |  |  |  |  |  |
| **4. Age** | .05 | .74*** | -.04 | - |  |  |  |  |  |  |  |
| **5. Standardized assessments** | .06 | .00 | -.01 | -.09* | - |  |  |  |  |  |  |
| **6. Interest in AI** | -.05 | .07 | -.02 | .07 | .11** | - |  |  |  |  |  |
| **7. Assessment time (hours)** | .01 | .02 | .01 | -.04 | .06 | -.07 | - |  |  |  |  |
| **8. Method: Unstructured** | .04 | .09* | .12** | .04 | -.13** | .01 | -.05 | - |  |  |  |
| **9. Method: Semi-structured** | .11** | -.05 | .04 | -.09* | .06 | .04 | -.04 | -.26*** | - |  |  |
| **10. Method: Structured** | -.08 | -.08 | -.09* | -.08 | .14** | .06 | .05 | -.13** | .22*** | - |  |
| **11. Method: Rating Scale** | .17*** | .05 | .13** | .04 | .20*** | .17*** | .03 | .02 | .14*** | .06 | - |
| **12. Method: Other** | .04 | .04 | .03 | .05 | .02 | .05 | .01 | .11* | -.04 | .02 | .08 |
| *Notes.* Significant codes: * = *p* < .05; ** = *p* < .01, *** *p* < .001.  Standardized assessments = Agreement with the importance of using standardized assessment methods for assessing depression (1 = *Strongly disagree* to 7 = *Strongly agree*)  Interest in AI = Interest in receiving AI decision support for assessing depression (1 = *Definitely not*, 5 = *Definitely*). | | | | | | | | | | | |

## **Follow up**

Figure S6 shows how the vast majority of clinicians follow up with the majority of their patients, with 68% following up on 60% or more, while 45% follow up with all of their patients, with “100%” being the most frequently reported percentage (mode = 100%) after the initial assessment. However, a significant portion (16%) report they never follow up with any of their patients.

The frequency of different assessment methods used for follow-up generally mirrors their usage during the initial assessment. Notably, unstructured interviews and rating scales/questionnaires were used at similar rates for the initial assessment (83% each). However, unstructured interviews are used more frequently for follow-up (65%) compared to rating scales/questionnaires (51%). The use of semi-structured interviews is reported at 22%, structured interviews at 11%, and other methods at 7%.

A) **Number (percentage) of patients that clinicians follow-up**

B) **The specific methods used for the follow-ups (although not necessarily for each patient)**

**Figure S6 A-B | Methods of Follow-up Assessments: A) Percentage of patients that clinicians follow-up, and B) Specific methods used for follow-ups (although not necessarily for each patient).**
Question: A) *How often (if at all) do you follow up on the patients’ development during the course of a depression treatment? n = 581 B) What method(s) do you use, and how often do you use them during the follow-up when treating depression? n = 581.*

## **Survey Questionnaire**

An English, Swedish, and Dutch version of the complete survey can be found in the open material: <https://osf.io/pw8mk/>

**INTRODUCTION**

**How often do you work with identifying, assessing and/or treating depression in a clinical setting?**

( ) I have never worked with identifying, assessing and/or treating depression in a clinical setting.

( ) I previously worked with identifying, assessing and/or treating depression in a clinical setting, but not anymore

( ) Once a month, or less

( ) Once a week, or less

( ) Several times a week

( ) Daily

**CONFIDENCE AND EXPERIENCE**

**How confident do you feel in identifying depression?**

( ) **0** Not at all confident

( ) **1** A little confident

( ) **2** Somewhat confident

( ) **3** Confident

( ) **4** Very confident

( ) **5** Extremely confident

**How many years have you had a job that includes identifying, assessing and/or treating depression?**

Only integer values may be entered in these fields.

year(s):	[	]

month(s):	[	]

**ASSESSMENT**

**On average, approximately how much time do you spend on identifying depression for a typical patient showing signs of depression or low mood?**

hour(s):	[	]

minutes(s):	[	]

**What method(s) do you use and how often do you use them when identifying, assessing, and/or excluding (ruling out) depression?**

*0% means that you never use the method; 50% means that you use the method on half of all your patients; and 100% means that you use the method for all your patients showing signs of depression or low mood.*

*You can use several methods for one patient (e.g., using both unstructured interview and rating scales to collect information).*

I use unstructured interviews for:	[	] % of the patients

(using clinical experience, but no instruments)

I use semi-structured interviews for:	[	] % of the patients

(predefined questions, allows follow-up questions)

I use structured Interviews for:	[	] % of the patients

(predefined questions, does not allow follow-up questions)

I use rating scales / questionnaires for:	[	] % of the patients

I use other method(s) for:	[	] % of the patients

**5.1) Which semi-structured interview(s) do you use for assessing depression?**

**5.2) Which structured interview(s) do you use for assessing depression?**

**5.3) Which rating scale(s) do you use for assessing depression?**

**5.4) Which other method(s) do you use for assessing depression?**

**[	]**

**How do you typically weigh the gathered information from different sources when assessing depression?**

Choose one of the following answers

( ) The final assessment (e.g., the final diagnosis or decision) is based on my clinical experience; that is, I weigh the various sources of information (e.g., the results from rating scales, interviews and conversations) in my head.

( ) The final assessment (e.g., the final diagnosis or decision) is based on a validated statistical model; that is, a statistical model (algorithm) weights the various sources of information.

( ) I do not weigh various sources of information, because I only use one method (e.g., the cut-off of a rating scale, or the outcome from a structured interview).

( ) Other:	[	]

**FOLLOW-UP**

**How often (if at all) do you follow up the patients’ development during the course of a depression treatment?**

*0% means that you never use the method; 50% means that you use the method on half of all your patients, and 100% means that you use the method for all your patients when assessing the development of patients showing signs of depression or low mood.*

*You can use several methods for one patient (e.g., using both unstructured interview and rating scales to collect information).*

Your answer must be between 0 and 100

Only integer values may be entered in these fields.

I use unstructured interviews for	[	] % of the patients

(using clinical experience, but no instruments)

I use semi-structured interviews for:	[	] % of the patients

(predefined questions, allows follow-up questions)

I use structured interviews for:	[	] % of the patients

(predefined questions, does not allow follow-up questions)

I use rating scales / questionnaires, for:	[	] % of the patients

I use other method(s), for:	[	] % of the patients

**7.1) What method(s) do you use and how often do you use them during the follow-up when treating depression?**

**7.2) Which semi-structured interview(s) do you use during the follow-up when treating depression?**

**7.3) Which structured interview(s) do you use during the follow-up when treating depression?**

**7.4) Which rating scale(s) do you use during the follow-up when treating depression?**

**7.5) Which other method(s) do you use during the follow-up when treating depression?**

**ATTITUDE**

**Indicate your level of agreement with the following statement:**

***‘When assessing depression, it is important to use standardized assessment methods that are demonstrated in research to have high validity^1^ and reliability^2^.’***

*^1^The degree to which the assessment method measures what it is supposed to measure.*

*^2^The degree to which the assessment method produces similar results under similar conditions.*

( ) **1** *Strongly disagree*

( ) ***2*** *Disagree*

( ) ***3*** *Somewhat disagree*

( ) ***4*** *Neutral*

( ) ***5*** *Somewhat agree*

( ) ***6*** *Agree*

( ) ***7*** *Strongly Agree*

Feel free to expand upon your answer above:	[	]

**ARTIFICIAL INTELLIGENCE**

**If given the opportunity, would you be interested in receiving decision support from Artificial Intelligence (AI) when assessing depression?**

**Condition 1**:

**Condition 2**: *Assume here that an AI-based decision support would meet the same or higher level of validity and reliability as current established psychometric tools (e.g., rating scales, questionnaires, structured interviews).*

**Condition 3**: *AI-technology has been shown to be able to provide highly accurate assessments of patients’ mental health. This is achieved using statistical methods that capture empirically established relationships between various sources of information (symptoms, situation, demographics etc) and diagnoses. The results from AI have been shown to have a higher level of accuracy compared to the assessments by clinicians using unstructured clinical interviews as their main assessment method. Research has since the 1950’s, supported its superiority in key areas of mental health assessment – and recently there has been an increase in research providing evidence of its benefits.*

( ) **1** *Definitely*

( ) **2** *Probably*

( ) **3** *Do not know*

( ) **4** *Probably not*

( ) **5** *Definitely not*

( ) *I have too little knowledge to be able to take a stand.*

**Feel free to develop your answer above:	[	]**

**ACCURACY**

**Based on your description of how you usually assess depression, how accurate do you believe your assessment is in comparison with the methods below?**

*Participants indicated their accuracy on a likert-scale (

***1*** *Much less accurate,* ***2*** *Less accurate,* ***3*** *Slightly less accurate,* ***4*** *Equally accurate,* ***5*** *Slightly more accurate,* ***6*** *More accurate,* ***7*** *Much more accurate****)*** ***for each of the following questions:***

Compared to other clinicians only using unstructured clinical interviews, my assessment is

Compared to other clinicians using a combination of sources, my assessment is

Compared to only using semi-structured interviews, my assessment is

Compared to only using structured interviews, my assessment is

Compared to only using rating scales, my assessment is

Compared to only using Artificial Intelligence, my assessment is

**DEMOGRAPHICS**

**How old are you?**

years:	[	]

**What gender do you identify yourself with?**

( ) Female

( ) Male

( ) Other	[	]

( ) No answer

**In which country do you work?**

( ) Sweden

( ) The Netherlands

( ) The United States

( ) Other	[	]

**13.1) In which region do you work?**

**13.2) In which province do you work?**

**13.3) In which state do you work?**

**Where do you work?**

[ ] Primary care: general practice / primary care center

[ ] Psychiatry department at a medical center

[ ] Mental/behavioral health practice at a medical center

[ ] Mental/behavioral health private practice

[ ] Other [	]

**How do you see patients?**

( ) I see all patients in person

( ) I See all patients digitally / via telehealth

( ) I do a mix of in-person and digital / telehealth care

**What is your job position?**

[ ] Psychiatrist (MD or DO)

[ ] Psychologist (PhD or PsyD)

[ ] Psychotherapist (Masters level)

[ ] Occupational therapist

[ ] Physician / general practitioner

[ ] Nurse

[ ] Other [	]

**Are you mainly working in the:**

[ ] Public sector

[ ] Private sector in an organization with less than 10 employees

[ ] Private sector in an organization with 10 – 100 employees

[ ] Private sector in an organization with more than 100 employees

[ ] Other [	]

**ENDING**

**How did you hear about this study?**

( ) Facebook

( ) LinkedIn

( ) Email

( ) Colleagues

( ) Other [	]

## **References**

Achenbach, T. M. (2011). Child Behavior Checklist. In J. S. Kreutzer, J. DeLuca, & B. Caplan (Eds.), *Encyclopedia of Clinical Neuropsychology* (pp. 123–126). Springer. <https://doi.org/10.1007/978-0-387-79948-3_1529>

Beck, A. T., Ward, C. H., Mendelson, M., Mock, J., & Erbaugh, J. (1961). An inventory for measuring depression. *Archives of General Psychiatry, 4*, 561–571. <https://doi.org/10.1001/archpsyc.1961.01710120031004>

Endicott, J., & Spitzer, R. L. (1978). A diagnostic interview: The Schedule for Affective Disorders and Schizophrenia. *Archives of General Psychiatry, 35*(7), 837–844. <https://doi.org/10.1001/archpsyc.1978.01770310043002>

First, M. B. (2015). Structured Clinical Interview for the DSM (SCID). In R. L. Cautin & S. O. Lilienfeld (Eds.), *The Encyclopedia of Clinical Psychology*. <https://doi.org/10.1002/9781118625392.wbecp351>

Grisham, J. R., Brown, T. A., & Campbell, L. A. (2004). The Anxiety Disorders Interview Schedule for DSM-IV (ADIS-IV). In M. J. Hilsenroth & D. L. Segal (Eds.), *Comprehensive Handbook of Psychological Assessment* (Vol. 2, Personality Assessment, pp. 163–177). John Wiley & Sons.

Hamilton, M. (1960). A rating scale for depression. *Journal of Neurology, Neurosurgery, and Psychiatry, 23*(1), 56–62. <https://doi.org/10.1136/jnnp.23.1.56>

Kaufman, J., Birmaher, B., Brent, D., Rao, U., Flynn, C., Moreci, P., Williamson, D., & Ryan, N. (1997). Schedule for Affective Disorders and Schizophrenia for School-Age Children–Present and Lifetime Version (K-SADS-PL): Initial reliability and validity data. *Journal of the American Academy of Child and Adolescent Psychiatry, 36*(7), 980–988. <https://doi.org/10.1097/00004583-199707000-00021>

Kovacs, M. (1985). The Children's Depression Inventory (CDI). *Psychopharmacology Bulletin, 21*(4), 995–998.

Kroenke, K., & Spitzer, R. L. (2002). The PHQ-9: A new depression diagnostic and severity measure. *Psychiatric Annals, 32*(9), 509–515. <https://doi.org/10.3928/0048-5713-20020901-06>

Lovibond, P. F., & Lovibond, S. H. (1995). Depression Anxiety and Stress Scales (DASS-42) [Database record]. APA PsycTests. <https://doi.org/10.1037/t39835-000>

Montgomery, S. A., & Åsberg, M. (1979). A new depression scale designed to be sensitive to change. *The British Journal of Psychiatry, 134*, 382–389. <https://doi.org/10.1192/bjp.134.4.382>

Poznanski, E. O., Cook, S., & Carroll, B. J. (1979). A depression rating scale for children. *Pediatrics, 64*(4), 442–450.

Radloff, L. S. (1977). The CES-D scale: A self-report depression scale for research in the general population. *Applied Psychological Measurement, 1*(3), 385–401. <https://doi.org/10.1177/014662167700100306>

Robins, L. N., Cottler, L. B., Bucholz, K. K., Compton, W. M., North, C. S., & Rourke, K. M. (2000). The Diagnostic Interview Schedule for DSM-IV (DIS-IV). Washington University School of Medicine.

Robins, L. N., Wing, J., Wittchen, H. U., Helzer, J. E., Babor, T. F., Burke, J., et al. (1988). Composite International Diagnostic Interview (CIDI, WHO CIDI, WHO WMH-CIDI) [Database record]. APA PsycTests. <https://doi.org/10.1037/t02121-000>

Rush, A. J., Giles, D. E., Schlesser, M. A., Fulton, C. L., Weissenburger, J., & Burns, C. (1986). The Inventory for Depressive Symptomatology (IDS): Preliminary findings. *Psychiatry Research, 18*(1), 65–87. <https://doi.org/10.1016/0165-1781(86)90060-0>

Rush, A. J., Trivedi, M. H., Ibrahim, H. M., Carmody, T. J., Arnow, B., Klein, D. N., et al. (2003). The 16-Item Quick Inventory of Depressive Symptomatology (QIDS), clinician rating (QIDS-C), and self-report (QIDS-SR): A psychometric evaluation in patients with chronic major depression. *Biological Psychiatry, 54*(5), 573–583. <https://doi.org/10.1016/S0006-3223(02)01866-8>

Sheehan, D. V., Lecrubier, Y., Sheehan, K. H., Amorim, P., Janavs, J., Weiller, E., et al. (1998). The Mini-International Neuropsychiatric Interview (M.I.N.I.): The development and validation of a structured diagnostic psychiatric interview for DSM-IV and ICD-10. *Journal of Clinical Psychiatry, 59*(Suppl 20), 22–57.

Tolin, D. F., Gilliam, C., Wootton, B. M., Bowe, W., Bragdon, L. B., Davis, E., et al. (2018). Psychometric properties of a structured diagnostic interview for DSM-5 anxiety, mood, and obsessive-compulsive and related disorders. *Assessment, 25*(1), 3–13. <https://doi.org/10.1177/1073191116638410>

Wing, J. K., Babor, T., Brugha, T., Burke, J., Cooper, J. E., Giel, R., et al. (1990). SCAN: Schedules for Clinical Assessment in Neuropsychiatry. *Archives of General Psychiatry, 47*(6), 589–593.

Yesavage, J. A., Brink, T. L., Rose, T. L., Lum, O., Huang, V., Adey, M., & Leirer, V. O. (1982). Development and validation of a geriatric depression screening scale: A preliminary report. *Journal of Psychiatric Research, 17*(1), 37–49. <https://doi.org/10.1016/0022-3956(82)90033-4>
